# Understanding Australian healthcare workers’ uptake of influenza vaccination: examination of public hospital policies and procedures

**DOI:** 10.1186/1472-6963-12-325

**Published:** 2012-09-19

**Authors:** Holly Seale, Rajneesh Kaur, C Raina MacIntyre

**Affiliations:** 1 School of Public Health and Community Medicine, Faculty of Medicine, University of New South Wales, New South Wales, Australia; 2 National Centre for Immunization Research and Surveillance of Vaccine Preventable Diseases (NCIRS), The Children’s Hospital at Westmead, New South Wales, Australia

**Keywords:** Influenza vaccination, Hospital, Healthcare worker, Policy

## Abstract

**Background:**

In Australia, whether to provide free influenza vaccine to health care workers (HCWs) is a policy decision for each hospital or jurisdiction, and is therefore not uniform across the country. This study explored hospital policies and practices regarding occupational influenza vaccination of HCWs in Australia.

**Methods:**

A study using qualitative methodology, which included semi-structured interviews, was undertaken with hospital staff involved with the delivery of occupational influenza vaccination from three states in Australia.

**Results:**

The 29 participants were responsible for vaccinating staff in 82 hospitals. Major themes in the responses were the lack of resources and the difficulties participants faced in procuring any additional support or funding from their institutions. All study sites provided vaccine free of charge to employees via on-site clinics or mobile carts, and used multiple strategies to inform and educate their staff. In some instances, declination forms had been adopted, however their use was associated with resourcing issues, animosity, and other problems. Participants who were responsible for multiple sites were more likely to recount lower vaccination coverage figures at their hospitals.

**Conclusions:**

From these interviews, it is clear that hospitals are implementing multiple strategies to educate, promote, and deliver the vaccine to staff. However, resources and support are not always available to assist with the vaccination campaign. The reality for many hospitals is that there is limited capacity to implement the vaccination campaigns at the levels high enough to raise compliance rates. Further research needs to be conducted to quantify the factors contributing to higher uptake in the Australian hospital setting.

## Background

As documented in the *Australian Immunisation Handbook – 9th Edition*[1], all healthcare workers (HCWs), including all staff and students directly involved in patient care or the handling of human tissues, are recommended to have the influenza vaccination. These recommendations are consistent with those made by the World Health Organization (WHO), the National Advisory Committee on Immunization (NACI), and the United States Center for Disease Control and Prevention (CDC) [2-5].

Although the vaccination of HCWs is a high priority everywhere, previous studies have shown that there are substantial variations in rates of influenza vaccine use between different countries [3,6-9]. There have been numerous studies conducted examining why people refuse to accept the influenza vaccine. Most choose not be vaccinated for a variety of reasons, including fear of needles, a belief that the disease is not serious, low risk perceptions, and a fear of side effects or adverse reaction [10-13]. In general, the reasons HCWs give for not being vaccinated are similar to those given by members of the general public. Hollmeyer et al. [14] undertook a systematic review looking at the predictive factors for influenza vaccination among HCWs. From the studies, they identified two major reasons for the lack of vaccine uptake by HCWs: first, a wide range of misconceptions or lack of knowledge about influenza infection; and second, a lack of convenient access to vaccine.

In Australia, provision of influenza vaccination to HCWs is a policy decision for each hospital or jurisdiction, and is therefore not uniform across the country. There have been individual studies of influenza vaccination policies and practices, but no clear snapshot of the policies and practices in the Australian hospital sector as a whole. From a previous survey of hospital based infection control and occupational health coordinators, it was reported that 76% (138 of 182) of the public hospitals surveyed, and 46% (36 of 79) of the private hospitals surveyed, provided annual influenza vaccination to their staff [15]. However, this study was conducted in only one state of Australia, and was undertaken over ten years ago. To examine the current situation, we used qualitative methods to explore the occupational influenza vaccination policies and practices currently in place in public hospitals in three Australian states.

## Methods

### Study design

Twenty-nine semi-structured interviews were completed with staff involved with the delivery of occupational influenza vaccination in public hospitals in three Australian states between June and August 2011. This approach was selected so that we could explore the processes and practices in place in these hospital settings along with the experiences of the staff responsible for vaccinating hospital employees. The study was approved by the Human Research Ethics Committee at the University of New South Wales (Reference: 2011-7-13).

### Participants

The study was open to all metropolitan public hospitals (including tertiary, principal referral, specialist women and children’s hospitals) from three states of Australia: New South Wales (NSW), Victoria (VIC), and South Australia (SA). Ninety-two public hospitals were identified as being eligible, based on information freely available on the websites of the state health departments (52 hospitals in NSW, 16 in SA, and 24 in VIC). The median numbers of overnight admissions for the hospitals represented in the study included were 8520 for NSW, 3914 for VIC, and 4824 for SA.

At each site, we identified a staff member (infection control coordinator, clinical nurse consultant, or nurse manager) who was responsible for coordinating the staff influenza vaccination campaign. The rationale for selecting these staff members to interview was that we believed they were in the best position to comment on the process, barriers, and problems associated with the campaigns. An invitation letter, consent form, and copy of the interview guide were sent to these stakeholders at each of the hospitals. Informed consent was collected from every participant prior to the interview. In some settings, one participant had responsibility for vaccinating staff at multiple hospitals.

### Data collection

Semi-structured, one-on-one interviews were conducted with the aim of exploring current occupational vaccination policies and the measures used to promote and distribute the influenza vaccine. Two of the investigators (HS and RK) collaboratively developed an interview guide. A conceptual or theoretical framework was not used in this study.

Questions were shaped to cover the key areas of interest, which included: (a) descriptive information (size of hospital, population served etc.); (b) hospital policies on annual influenza vaccinations for employees; (c) implementation practices (use of clinics, mobile carts, etc., the use of signed declination forms for those refusing vaccination; consequences for not following the requirements); (d) activities taken to encourage immunizations (incentives, promotion and education); and (e) modes of storing data on HCW vaccinations (use of software, databases, etc.). Participants were also asked to comment on levels of staff vaccination. Last, we explored the potential barriers to employee vaccinations and institutional and staff support for a policy of mandatory annual influenza vaccinations. Pre-designed prompts were employed throughout the interview to trigger interviewees’ thoughts. Interviews were conducted by HS and RK and lasted up to 45 minutes. Each facility was called up to five times before it was considered a non-respondent.

### Data analysis

The interviews were digitally recorded, transcribed verbatim, and analysed thematically. Two investigators (HS and RK) developed a list of themes after one quarter of the transcripts had been analysed. An agreed framework was then applied to another subsample of transcripts and modified further. Using this final framework, all of the transcripts were analysed and coded. Text was organised within the identified themes of the developed framework without the use of any software. No formal testing of the reliability of the coding was undertaken, although discussions with colleagues about the analysis and the meanings and patterns derived from it were extensively undertaken. Data on the number of hospital beds at each site was extracted from the Australian federal government’s my hospitals website ( http://www.myhospitals.gov.au/).

## Results

Overall, 29 of the 34 invited stakeholders (85.3%) participated in the study. Three identified stakeholders refused to participate and another two could not be contacted. The remaining 29 participants were responsible for or involved with the delivery of occupational influenza vaccination in 82 responding hospitals out of 92 hospitals of interest from the three states. These hospitals were classified, as per the Australian government’s myhospitals website, into the following categories: 54 general, 25 tertiary, and 3 specialist. Sixteen participants represented single sites, 8 represented 2–5 sites, 3 represented 6–9 sites, and 2 represented more than 9 sites. The vaccine was provided freely to employees at all sites.

### Structure and resources associated with the campaigns

When asked about the existence of a formal written policy regarding employee influenza vaccination, a reference was generally made to a policy published by the state health department, which states that the uptake of the influenza vaccine by HCWs is “highly recommended.” Only one participant reported that influenza vaccination of the staff at their site is “essential,” and that their site requires employees who refuse vaccination for any reason to discuss with their manager the risk of being unvaccinated and of possible exposure to a patient with influenza. At this site, any staff member who declines to receive the influenza vaccine (because of an allergy, illness, or non-medical reason) is required to complete a declination form.

In order to make the influenza vaccine easily accessible at their hospitals, numerous strategies to deliver the vaccine have been utilised. These range from having permanent “drop in” staff health clinics to temporary clinics (within wards or the staff cafeteria) and using mobile carts. However, not all sites have the capacity to run a fixed clinic; those that do not rely on mobile carts to deliver the vaccine. At one hospital, the wards are responsible for vaccinating their own staff, while at other sites, temporary vaccine clinics are set up to vaccinate staff *en masse* over two days. The importance of using mobile carts was highlighted, as it was felt that going to the staff members in their own settings indirectly placed “pressure” on them, which resulted in higher uptake rates.

"We run two 12-hour flu vaccination days over two days. … We have five to six vaccination stations and a triage section … We have people checking the consents and then people just flow through so it’s very, very quick when we have, you know, six vaccination stations going and they have their vaccine and then go back to work. (Tertiary hospital, 500 beds)."

In most instances, the vaccine campaigns also target non-clinical staff such as volunteers, students, laboratory staff, ancillary staff, and the health service childcare centre staff.

### Staffing and resource issues

The need for surge staff to assist with the initial weeks of the campaign was not uncommon, while in other instances staff had to be “rationed” so that all sites were serviced. The capacity to increase the number of clinics run or the hours they are open for was deemed to be limited because of these staffing constraints.

"I think we would probably get higher compliance if we had more time, like we can only spend so much time…(Tertiary hospital, 200–500 beds)."

Aside from being responsible for organising general staff health activities, staffing the vaccine clinics, and promoting and educating, participants are also involved in other infection control activities, such as exposure management. The vastness or relentlessness of the “task” each year was often mentioned, while the lack of support or resources available from within the hospital setting was highlighted. One participant went as far as to say that during “the six weeks of the flu campaign, all of our other work goes on hold.”

"It’s hard work given it’s quite a negative environment and poorly resourced.(Tertiary hospital, 100–200 beds)."

It was not uncommon for participants to spread their time over multiple sites, with one participant even reporting that they are responsible for staff vaccination at 19 sites. Participants who estimated that their coverage rates were above 50% were generally only responsible for a single site, and often commented on the considerable amount of resources available to them.

### Informing and educating staff

The need to continually promote the importance of getting vaccinated at every opportunity was a common theme, with many participants relying on the resources produced by the drug companies to promote the vaccine. A wide range of methods were utilised to inform staff about the availability and need for influenza vaccination, with multiple strategies often being adopted. Emails were the most common method, followed by newsletters or flyers. Other approaches mentioned included posters, payslips, pop-up screen reminders, letters from hospital directors, and setting up booths in the cafeteria. Some indicated that they provided information and education at orientation days and at grand rounds (medical and nursing).

"As we see people, we’ll say “we’ll see you next year at flu season time”, and if there’s any reluctance then we’ll discuss matters with them out of season. The education is ongoing. (Tertiary hospital, >500 beds)"

"We put posters around everywhere. I sent out letters to all heads of department, and you know, and these are all round the hospital, but it doesn’t seem to make any difference, really, for the uptake. The only time the uptake really increases is if there’s a, like swine flu, like in bird flu, SARS, you know, like when SARS was around, when people are really scared, when the media kind of starts to kind of scare everybody to come. (Tertiary hospital, >500 beds)."

Less common approaches included providing an eight-minute DVD to staff, produced by one of the main vaccine distributors, which challenges staff to consider whether “they can afford the flu.” Last, one group highlighted that they had instigated an “ask the expert” helpline so that staff members could get their questions about the disease and the vaccine answered by a medical staff member located at the hospital.

"A letter goes out from the general manager as well as the director of clinical services, strongly advising that staff have the vaccination. We present at grand rounds, the medical grand rounds and nursing grand rounds. There’s an email reminder and flyers and we’ve tried an “ask the expert” kind of strategy so people that have got questions about flu vaccination ring someone that is an expert in that so we have a doctor that agreed to participate in that. It’s in all the newsletters and all that kind of stuff. (Tertiary hospital, 500 beds)."

"As soon as the vaccine arrives, they start walking around a lot with their high visibility vests on that have got the flu bug on the back. (General hospital, 200–500 beds)"

### Motivators driving vaccination

The use of incentives was a common factor, with many relying on the products provided by the vaccine manufacturer (often a lollipop). Participants highlighted that it was common for staff not to turn up at the vaccine clinic if they found out the incentives were not available or had run out. Not all incentives were targeted at the individual, with some sites awarding a monetary prize (partly donated by the vaccine manufacturer) to the ward that had the highest overall levels of influenza coverage. However, the ability to routinely provide this type of incentive was not ubiquitous. In many instances the hospitals were unable or reluctant to provide any incentives, with one participant saying that they had used gifts donated by volunteers, and unwanted “Christmas” presents, to make up raffle prizes, or had dipped into their own department budget to buy chocolates as incentives. Due to funding issues, most of the perks (such as morning teas for staff during the vaccine clinics) had ceased.

"If we didn’t actually have the lollipop I think we’d have less people being vaccinated, because whenever we run out there’s a mass walkout. (Tertiary hospital, >500 beds)."

"Can we get a coffee thing to get people to come down? There’s no money in it. The hospitals have no money and … so nothing more than giving the lollipop works. (Tertiary hospital, 100-200 beds)."

The positive effect of having champions within the hospital system actively promoting the need for staff to get vaccinated, or “rolling up their sleeve” and getting vaccinated as an example, was spoken about. On the flipside, others commented on the negative impact of having managers or senior hospital staff who did not support influenza vaccination.

"We lost our CEO … he was an absolute wonderful, wonderful support. Every year he used to have his photo taken whilst we gave him his flu vaccine … But this year, he actually left at the end of last year, and our new (CEO) … they just weren’t interested. They didn’t want to do it. (General hospital, 200–500 beds)"

"There are a lot of clinical people and people in senior clinical roles who are anti influenza vaccination, which just amazes me … We have one in (a high risk) department and it’s a very senior level, and that person loves to tell them all every year it’s a waste of time. You know, it’s just gonna make you sick. You’re gonna get the flu from it, you’re gonna be ill. (General hospital, 200–500 beds)."

While declination forms had been implemented in some sites, the practice was deemed to be resource intensive and associated with staff animosity and other problems. Participants believed that the problems stemmed from the fact that receiving the vaccine was not “mandatory,” so there was no governance behind the action. They could not “force” the staff members to complete the form, and too many thought it was a “pointless activity in the end,” and had abandoned the use at most sites within a season.

"We have tried to do that in the past. This year we didn’t because it always creates a lot of animosity. (General hospital, 100–200 beds)."

"Yeah, it was a problem in terms of staff relations. They felt they were being forced to do something they didn’t believe in and yeah, so we haven’t used a declaration, an opt-out policy. Not again anyway. (Tertiary hospital, >500 beds)."

"You need more staff members … You need extra resources for all of this and for arguing and if you had to explain why they didn’t have it … you’d just need more resources. Until people fund this, it’s not possible. (Tertiary hospital, 100–200 beds)."

### Difficulties of keeping vaccination records

While some form of record keeping was done at most sites to document on-site staff influenza vaccination, not all hospitals had the capacity to maintain electronic databases and instead relied on hard copy consent forms as evidence of uptake. Many commented on the enormity of the task of manual entry for the coverage data each year. Generally, there were no organised mechanisms for recording off-site vaccination or for keeping records for personnel not directly employed by the hospital (agency staff, volunteers, students, etc.) due to resource constraints and feasibility issues.

"It’s an extremely resource intensive job. So you know, if you’re looking at 1,200 staff, putting that data into the database every year, we don’t have the resources to do it. (General hospital, 100–200 beds)."

"We did have an intranet database for the last two seasons, so we were able to have online graphs which drilled down to hospital, you know, department, and category of staff. That wasn’t able to be supported. It was linked to the payroll, so they actually gave an accurate record of who was vaccinated or who received vaccination from the staff clinic, but yeah, it wasn’t supported this year so we’re just back to doing our own by staff category, hospital graphs in an Excel kind of format. (Tertiary hospital, >500 beds)."

The problems associated with not having databases ranged from not being to track staff members who were unvaccinated through to not being able to identify wards and departments with overall low uptake rates. Participants spoke of the frustration of not being able to appropriately target areas with low uptake with the appropriate resources or education.

"It’s a bit difficult for us because the real denominator is not that accurate. We always get problems with getting the real denominator … staff are on leave, temporary staff, bank staff to filter through that. (Tertiary hospital, >500 beds)."

While the available vaccine coverage data (or estimates) were conveyed back to either the ward managers or the hospital board, the information was neither being strategically utilised as patient safety indicators nor being made available to staff.

### Future directions: need for a culture change

When asked to comment on whether they felt their hospital and/or staff would support a mandatory influenza vaccination policy, the response was extremely mixed. For the participants who stated that there would be outright hospital support, the main reason provided was that the policy would promote patient protection and would reduce personal risk and sick leave among staff. One participant felt that it was the only way to increase coverage figures among their staff. Others felt that it would be supported if the policy or directive came from the state health department. However, numerous comments were made about the problems associated with implementing this type of policy, with most remarking that the difficulties lay in not having enough infrastructure and resources to provide a vaccine that needs to be administered on a yearly basis. One participant stated that there needed to be a “culture change” around vaccination support before they could implement a mandatory vaccination policy.

"How can you make it compulsory? Are you saying people can’t start their shift? I just can’t imagine how, you know, I’d be standing at the front door, [saying], “You can’t go to your shift until I’ve jabbed you. (General hospital, 50–100 beds)."

When asked to comment on whether staff would support a mandatory vaccination policy, it was felt that there would be little support among staff. Some stated that they felt their staff would see it as a violation of their rights. However on the flipside, others felt that if there were consequences associated with not complying, or if the policy were instigated from the state or national health department, then it would be accepted and complied with.

"But you know, if they’re told, “well, you take it, or you don’t come to work,” they tend to take it. (Tertiary hospital, >500 beds)."

"I know the ones that will never have the influenza vaccine and I don’t think anything will change that, unless of course they are definitely told if you’re not going to have it, you can’t work. (Tertiary hospital, 50–100 beds)."

## Discussion

Using qualitative methods, this study explored the occupational influenza vaccination policies and practices currently in place in public hospitals in three states of Australia. In every interview, the central issue participants highlighted was the enormity of the task of promoting and delivering the vaccine annually, sometimes in an environment with extremely low staff support. The difficulty in procuring any resources or funding or extra staff members was also mentioned, and was perceived to affect the ability to sufficiently promote and deliver the vaccine to increase compliance rates. In some instances, participants were responsible for overseeing the delivery of the influenza vaccine to staff over a large number of sites (19 in one instance), or sites that are located in different parts of a city (separated by 50 km or more). Last, many sites did not have the capacity to properly document and maintain databases and track coverage.

The need to continually promote the importance of getting the influenza vaccine at every possible opportunity was a common theme in our study. Most sites employed multiple strategies to educate staff about the importance of getting vaccinated and to deliver the vaccine to staff. Mobile carts, out of hours clinics, and peer-to-peer vaccination were just some of the methods recounted.

Non-mandatory campaigns using these measures have previously been shown to be successful at increasing influenza vaccination among HCWs [16-18]. For example, Quan et al. reported an increase of 20% in the vaccination rate after the introduction of a program of strategies that included decentralised vaccine distribution, the use of mandatory declination forms, and the use of mobile carts to provide vaccines at routinely scheduled physician and medical staff meetings and directly to clinical units. While this pushed the vaccine coverage at the hospital to 62.9%, cumulative campaigns using these strategies did not result in any further changes to the rate [19]. In closing, the authors highlighted that even though the campaign significantly improved vaccination levels beyond the national norm, a mandatory vaccination policy was needed to reach vaccination levels in excess of 90% [19].

The use of incentives was common factor among the interviews. In most instances, the incentives were merely a lollipop that had been provided by the vaccine manufacturer. On some occasions, raffles or competitions had been used to encourage staff to get vaccinated. In these cases, sites had to use their own budgets or rely on gifts from other members of the hospital community to use as prizes, and hence were not able to maintain the practice. In 2005, Kimura et al. reported on the interventions used to increase influenza vaccination of HCWs in California and Minnesota [20]. After the introduction of an incentive (a movie ticket or books on health or medical topics), the site recorded a 13.8% increase in uptake among its employees. However, it is difficult to quantify the impact of the introduction of the incentive in this setting, as a range of other strategies was already in place at the sites.

While there are mixed reports about the impact of incentives on occupational vaccination, in other areas of employee health and safety the evidence is strong that incentives and rewards work better than punishments [21]. From our interviews, incentive giving appears to be an approach that participants feel is important to continue. Given the problems associated with providing each vaccinated staff member with an individual incentive, institutions may want to consider the use of public recognition for HCWs who choose to accept influenza immunization. For example, there could be honourable mentions or rewards for hospital units or wards whose staff vaccination rate reached a set percentage [22].

While the use of “opt out” declination forms was reported by a number of our respondents, most found the practice resource intensive and problematic, and had abandoned using them. The intent of a declination statement is to ensure that HCWs are appropriately informed of the rationale for influenza vaccination, to promote the message of patient safety, and to dispel commonly held misconceptions about influenza and influenza vaccination. The major benefit associated with using these forms is that, unlike the introduction of mandatory vaccination policies, they allow HCWs to retain their autonomy and their right to refuse treatment: they can simply choose to opt out if they do not wish to be vaccinated. However, to date most studies have failed to show any real or substantial benefit associated with their use [23].

What has been established is that the use of declination forms is associated with increased resources to track compliance (as noted by a number of our participants), the risk of negatively affecting the employer–employee relationship, and the need for institutions to determine the punitive consequences for HCWs who refuse to sign the document. In the concluding statement of a recent review examining the use and impact of declination statements the authors emphasised that there may be increases in vaccination coverage, and a decrease in staff misconceptions about the influenza vaccine, if the declination statements were bundled with other measures that emphasised the rationale for and importance of vaccination, and decreased barriers to receipt of the vaccine [17].

The difficulty in maintaining databases and tracking staff vaccination was often highlighted during the interviews. Some sites did not have the capacity to follow up with staff members who were vaccinated off-site, whiles others did not keep records for non-payroll hospital workers (volunteers, ancillary staff, and agency staff). At the extreme, some sites relied on hard copy consent forms as evidence of staff vaccination because they did not have the capacity to enter the information into a computerised system. As a result of this, participants spoke of the frustration of not being able to appropriately target areas with low uptake with resources and/or education.

Previous studies have recognised the benefits associated with the ability to build computer programs to calculate vaccination rates. These include: (1) easy identification of noncompliant staff members and the provision of information back to ward managers, (2) calculation of and feedback about rates throughout the influenza season, and (3) mobilising of resources or interventions to target groups within the hospital that continue to have low levels of uptake [24]. If healthcare institutions adopt a method for monitoring and reporting on influenza vaccination rates, fair and uniform comparisons of these rates across institutions could also be possible.

When respondents were asked whether their hospital and staff would support a mandatory influenza vaccination policy, a mixed response was received. It was felt that institutions would not independently introduce a policy, and that currently there was no support for it among staff. The perceived lack of support for a mandatory influenza vaccination policy correlates well with the findings from a survey of Australian HCWs [25], which reported that less than 50% of staff supported the inclusion of the influenza vaccine into a national policy. Participants did, however, acknowledge that a mandatory policy would be acceptable if it was executed by the state health department or if it included consequences for non-compliance (i.e. a policy of mask use or relocation). Further studies need to be conducted to explore the need for and use of mandatory policies in an Australian setting.

The high response rate and the use of in-depth interviews to elicit a greater depth in the information are two key strengths of the present study. Limitations include that (1) member checking of themes was not undertaken; and (2) interviews were only undertaken with a select group of hospital staff, so the possibility of other important themes emerging cannot be ruled out. Specific details regarding the participants’ role at the hospital was also not included. This was a small, qualitative study, and the findings should be explored further in larger, quantitative studies.

## Conclusions

From these interviews, it is clear that hospitals implement multiple strategies to educate, promote and deliver the vaccine to staff. However, resources and support are not always available to the campaigns. The reality for many hospitals is that there is limited capacity to implement these strategies more intensively to drive compliance rates higher. Further research needs to be conducted to identify the factors contributing to higher uptake in the Australian hospital setting.

## Competing interests

Dr Seale has received lecture fees from CSL and Sanofi Pasteur. Prof. C. Raina MacIntyre receives funding from influenza vaccine manufacturers GSK and CSL Biotherapies, for investigator-driven research. These payments were not associated with this study. Rajneesh Kaur declares that she has no competing interests.

## Authors’ contributions

HS and RK participated in the design of the study and interview guide, undertook the interviews, performed the analysis and drafted the manuscript. CRM reviewed the manuscript. All authors read and approved the final manuscript. The corresponding author had full access to all the data in the study, and had final responsibility for the decision to submit for publication.

## Pre-publication history

The pre-publication history for this paper can be accessed here:

http://www.biomedcentral.com/1472-6963/12/325/prepub
